# Productive and morphological responses of japanese quails (*Coturnix japonica*) supplemented with phytase superdosing at different temperatures

**DOI:** 10.1016/j.psj.2026.106508

**Published:** 2026-01-23

**Authors:** Raiane dos Santos Silva, Apolônio Gomes Ribeiro, Adiel Vieira de Lima, Paloma Eduarda Lopes de Souza, Edijanio Galdino da Silva, Isabelle Naemi Kaneko, Cleber Franklin Santos de Oliveira, Carlos Henrique do Nascimento, Dayane Albuquerque da Silva, Fernando Guilherme Perazzo da Costa, Edilson Paes Saraiva, Lucas Rannier Ribeiro Antonino Carvalho, Ricardo Romão Guerra

**Affiliations:** a Department of Animal Science, Federal University of Paraíba, Areia, Paraíba, Brazil; b Department of Veterinary Sciences, Federal University of Paraíba, Areia, Paraíba, Brazil; c Department of Animal Science, Federal University of Rondônia (UNIR), Brazil; d Department of Statistics, Federal University of Alfenas (UNIFAL-MG), Rua Gabriel Monteiro da Silva, 700, Centro, Alfenas – MG, Brazil; e Department of Animal Science, Universidade Federal Rural de Pernambuco, Recife, Pernambuco, Brazil; f Department of Physiology and Pharmacology, Karolinska Institutet, Biomedicum 5B, Solnavägen 9, S-171 77, Stockholm, Sweden

**Keywords:** Egg quality, Heat stress, Intestinal morphology, Japanese quail, Phytase superdosing

## Abstract

This study aimed to evaluate the effects of phytase superdosing in the diet of Japanese quails subjected to different thermal conditions on productive performance, egg characteristics, intestinal morphometry, and physiological parameters. The experiment was conducted in a completely randomized design in a 5 × 3 factorial arrangement, consisting of five phytase levels (0, 500, 1000, 1500, and 3000 FTU/kg) and three temperatures (24, 30, and 36°C), with six replicates of eight birds each. The performance variables evaluated included feed intake, egg production, egg weight, egg mass, feed conversion per egg mass, and feed conversion per dozen eggs. Egg quality was assessed by yolk color, shell thickness, specific gravity, Haugh units, and proportions of yolk, albumen, and shell. Spleen, liver, heart, and abdominal fat weights were also measured, along with duodenum and jejunum morphometry. Data were subjected to analysis of variance, Tukey’s test, and regression analysis. Birds kept at 36°C showed lower feed intake compared with those maintained at 24 and 30°C (*P* < 0.001), with no significant effect of phytase on performance. Supplementation with 1500 FTU increased eggshell thickness at 36°C (*P* < 0.001). A temperature × phytase interaction was observed for liver weight (*p* = 0.010), whereas heart weight was higher at 24°C compared with 36°C (*P* = 0.005). The effects of phytase on duodenal morphometry were temperature-dependent, with the greatest absorptive area observed with 3000 FTU at 30°C (*P* < 0.001). The temperature of 24°C resulted in the best morphological parameters and the lowest hepatic glycogen index. It is concluded that although quails tolerate temperatures of 36°C, the best productive responses occur at 30°C. Therefore, the use of 1000 to 1500 FTU is recommended, as these levels provided benefits to intestinal morphometry and egg quality.

## Introduction

Heat stress represents a major challenge for the poultry industry, drastically affecting the health, welfare, and productivity of birds (Mazzoni et al., 2022). It occurs when thermoregulation is impaired, meaning the animal accumulates or generates more heat than it can dissipate to the environment (Gonzalez-Rivas et al., 2020). Birds under heat stress experience changes ranging from behavioral alterations (such as reduced feed intake) to physiological disturbances, including structural damage to the intestinal epithelium. As a result, there is a severe decrease in nutrient digestibility and absorption (Rostagno et al., 2020; Ribeiro et al., 2024a,b; Ribeiro et al., 2025).

The impairment of intestinal integrity and digestive efficiency caused by heat stress increases the susceptibility of birds to dietary antinutritional factors, further compromising nutrient utilization. In this context, phytate (an antinutritional factor) further exacerbates the problem. It not only reduces phosphorus bioavailability but also interferes with the absorption of minerals, amino acids, and dietary energy (Bernardes et al., 2022; Dallmann et al., 2023). The development of nutritional strategies, such as the use of phytase, aims to improve the welfare of birds exposed to high temperatures (Lima et al., 2025). Phytase, an exogenous enzyme, is nutritionally beneficial for hydrolyzing phytic acid present in feeds, releasing phosphorus, calcium, and other nutrients bound to phytic acid. This process increases nutrient bioavailability and reduces the antinutritional effects of phytate (Cowieson et al., 2006, Cowieson et al., 2011; Sena et al., 2020).

Phytase doses above commercial recommendations, commonly referred to as phytase superdosing, have attracted increasing scientific interest due to their ability to promote faster and more complete degradation of phytate, thereby maximizing nutrient release (Farias et al., 2021). In general, superdosing involves the inclusion of phytase at levels two to three times higher than conventional recommendations (500FTU de Phytase), which enhances the availability of phosphorus and other nutrients bound to the phytate complex (Ribeiro et al., 2024b). Studies have consistently reported beneficial effects of phytase superdosing in poultry, including improvements in productive performance (Sampath et al., 2023), amino acid digestibility (Dersjant-Li et al., 2022), and eggshell quality (Farias et al., 2021). It should be emphasized that the majority of studies investigating phytase superdosing have been conducted in laying hens and broilers, mainly under thermoneutral environmental conditions. Although these findings provide robust evidence of the nutritional and physiological benefits of high phytase inclusion, their applicability to other poultry species and to challenging thermal environments remains limited. This gap underscores the need for targeted investigations evaluating the efficacy of phytase superdosing under heat stress conditions, particularly in laying Japanese quails.

Therefore, the present study aimed to evaluate the effects of phytase superdosing in diets of laying Japanese quails exposed to potential heat stress, considering production data as well as morphological characteristics of the reproductive and digestive systems.

## Materials and methods

The experimental trial was conducted in the climate-controlled chambers of the Research Unit for Bioclimatology, Behavior, and Animal Welfare, Department of Animal Science, at the Center for Agricultural Sciences of the Federal University of Paraíba, Campus II, in Areia, Paraíba, Brazil. The experimental protocol N°. 3695120121 was approved by the Animal Use Ethics Committee of the Federal University of Paraíba (CEUA-UFPB).

### Animals, facilities, and experimental design

A total of 720 Japanese quails in the production phase were used, starting at eight weeks of age with egg production above 95%. The birds were housed in climate-controlled chambers and distributed into 15 treatments with six replicates of eight birds each, in a completely randomized design in a 5 × 3 factorial arrangement, consisting of five phytase levels (0, 500, 1000, 1500, and 3000 FTU/kg) and three temperature ranges (24, 30, and 36°C), representing thermoneutrality (24°C) and heat-stress conditions (30 and 36°C). Within each bioclimatic chamber (temperature), all five phytase levels were applied, and experimental replicates consisted of cages (eight birds per cage), totaling six replicates per treatment within each temperature. The experiment was divided into five cycles of 21 days, with the quails in the laying phase, totaling 105 days of evaluation.

**Fig. 1:**
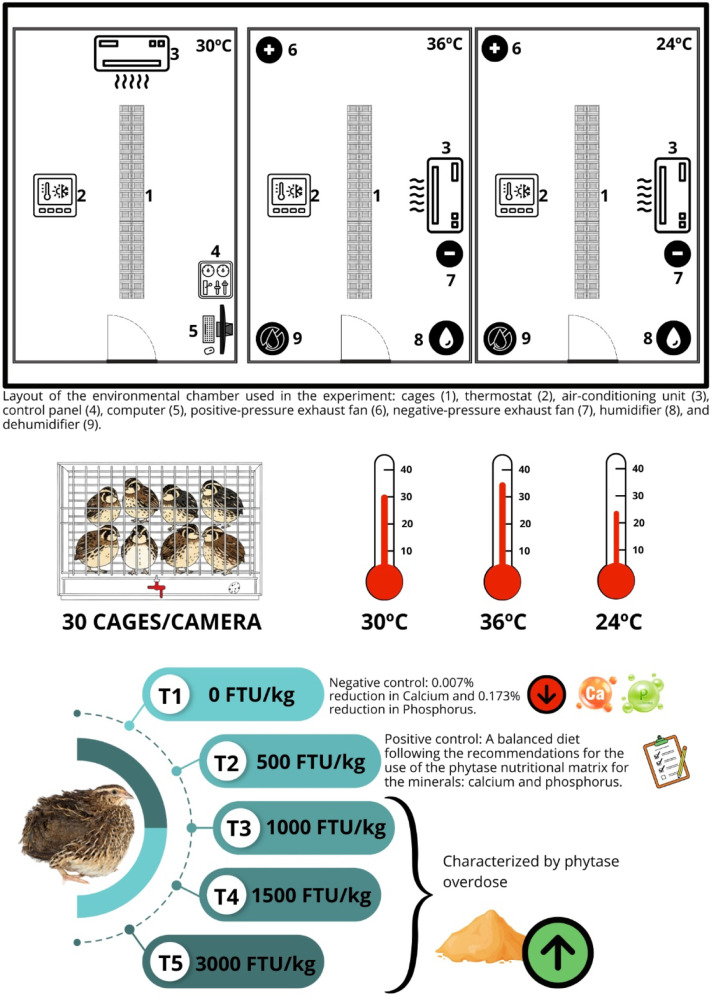
Schematic representation of the experimental design and period. three bioclimatic chambers were used, each maintained at a constant temperature (24, 30, or 36°C). within each chamber, all five phytase levels (0, 500, 1000, 1500, and 3000 FTU/kg) were applied, with cages as experimental units (eight birds per cage), totaling six replicates per treatment within each temperature.

Three bioclimatic chambers with an area of 19.71 m² were used, equipped with systems capable of achieving and maintaining the experimental temperatures of 24, 30, and 36°C. Each room measured 5.40 m in length and 3.83 m in width. In each chamber, 30 galvanized-wire cages (55 × 35 × 27 cm) were installed, equipped with trough-type feeders and nipple drinkers suitable for the bird’s production phase. The chambers were fitted with air-conditioning, heating, humidification and dehumidification systems, exhaust fans, thermal control via thermostats, and lighting systems (Fig. 1). Feeders were supplied with the experimental diets twice daily, at 7:00 a.m. and 4:00 p.m. Throughout the experimental period, birds had ad libitum access to feed and water. The lighting program used was 17 h/day of light throughout the experimental period.

### Experimental diets

The diets (Table 1) were formulated according to the recommendations of the Brazilian Tables for Poultry and Swine (Rostagno et al., 2017), differing only in phytase supplementation. All diets were formulated considering the nutrient release matrix provided by 500 FTU (0.165% Ca – 0.150% P). The basal diet was not supplemented with phytase. The other diets were supplemented with 500, 1000, and 3000 FTU/kg of feed, applied in an on-top approach by replacing the inert ingrediente (Fig. 1).

**Table 1:** Experimental diets with phytase supplementation for laying Japanese quails.

**Treatments**		**T1**	**T2**	**T3**	**T4**	**T5**
**Ingredientes**	**Unit**	**0FTU**	**500FTU**	**1000FTU**	**1500FTU**	**3000FTU**
Corn - 7,88%	g/kg	597	597	597	597	597
Soybean meal 45,22%	g/kg	305	305	305	305	305
Soybean oil	g/kg	6.67	6.67	6.67	6.67	6.67
DL-methionine	g/kg	3.98	3.98	3.98	3.98	3.98
L-Lysine HCl	g/kg	2.65	2.65	2.65	2.65	2.65
L-threonine	g/kg	0.35	0.35	0.35	0.35	0.35
Limestone	g/kg	74.37	74.37	74.37	74.37	74.37
Dicalcium Phosphate 18,5%	g/kg	4.00	4.00	4.00	4.00	4.00
Common Salt	g/kg	3.45	3.45	3.45	3.45	3.45
Mineral premix ^1^	g/kg	0.50	0.50	0.50	0.50	0.50
Vitamin premix ^2^	g/kg	0.25	0.25	0.25	0.25	0.25
Choline	g/kg	0.70	0.70	0.70	0.70	0.70
Antioxidante ^3^	g/kg	0.10	0.10	0.10	0.10	0.10
Inert ^4^	g/kg	0.60	0.50	0.40	0.30	0.00
Phytase ^5^	g/kg	0.00	0.10	0.20	0.30	0.60
Total		1000	1000	1000	1000	1000
**Nutrients**	**Unit**					
Phytase	FTU/kg	0	500	1000	1500	3000
Metabolizable energy	kcal/kg	2800	2800	2800	2800	2800
Crude protein	g/kg	190.00	190.00	190.00	190.00	190.00
Calcium	g/kg	29.93	29.93	29.93	29.93	29.93
Phosphorus total	g/kg	3.94	3.94	3.94	3.94	3.94
Available phosphorus	g/kg	1.77	1.77	1.77	1.77	1.77
Potassium	g/kg	7.32	7.32	7.32	7.32	7.32
Sodium	g/kg	1.55	1.55	1.55	1.55	1.55
Chlorine	g/kg	3.19	3.19	3.19	3.19	3.19
Mogin number	mEq/kg	164.59	164.59	164.59	164.59	164.59
Digestible amino acid (%)						
Digestible Methionine	g/kg	6.47	6.47	6.47	6.47	6.47
Digestible Methi. + cystine	g/kg	9.08	9.08	9.08	9.08	9.08
Digestible Lysine	g/kg	11.07	11.07	11.07	11.07	11.07
Digestible Threonine	g/kg	6.75	6.75	6.75	6.75	6.75
Digestible Tryptophan	g/kg	2.07	2.07	2.07	2.07	2.07
Digestible Valine	g/kg	7.98	7.98	7.98	7.98	7.98

The treatments mentioned above were subjected to three different temperatures (24, 30 and 36°C). ^1^ Mineral Premix (concentration/kg of product): Mn - 60 g, Fe - 80 g, Zn - 50 g, Cu - 10 g, Co - 2 g, I - 1 g and Se - 250 mg. ^2^ Vitamin Premix (concentration/kg of product): Vit. A - 15 mil UI, Vit. D3 - 1,500,000 UI. Vit. E - wm 15000; Vit.B1 - 2.0 g, Vit. B2 - 4.0 g Vit. B6 - 3.0 g, Vit. B12 - 0015 g, nicotinic acid - 25 g, pantothenic acid - 10 g; Vit.K3 - 3.0 g, folic acid - 1.0 g. ^3^Antioxidante = BHT (butil-hidroxi-tolueno). ^4^Inert = Kaolin. ^5^Phytase enzyme = 100 grams/ton provides 500 FTUs/kg of feed.

### Zootechnical variables

During the 15 days prior to the beginning of the experiment, complete plots of 8 birds were identified, and the total number of eggs produced was recorded daily to obtain baseline production averages. After this procedure, the birds were allocated according to their average productivity, and then the plots were weighed and identified by treatment and replicate.

The performance and egg quality variables evaluated were: feed intake (g/day), egg production (%), egg weight (g), egg mass (g/day), feed conversion per egg mass (kg/kg) and per dozen eggs (kg/dz), yolk color, shell thickness (mm), and the percentage (%) of yolk, albumen, and shell, as well as Haugh units and specific gravity (g/cm³).

The performance evaluation period was divided into five 21-day periods. At the end of each period, feed leftovers from each replicate were collected to calculate feed intake. Egg collection was carried out twice a day (08:00 and 15:00 h) and recorded on a production and mortality sheet to correct the data. Egg production (%) was calculated by dividing the total number of eggs per replicate by the number of birds, corrected for mortality when applicable.

Eggs from the last three days of each period were individually weighed to determine the average egg weight. Egg mass was calculated by multiplying egg production by the average egg weight per replicate. Feed conversion per egg mass was calculated as the ratio between feed intake and egg mass produced. Feed conversion per dozen eggs was calculated by dividing feed intake by egg production, and then multiplying the result by twelve.

At the end of each period, four eggs per replicate were selected to determine the weight and percentage of yolk, albumen, and shell after manual separation of the components. The percentage of each egg component was obtained by dividing the weight of the component by the egg weight and multiplying by 100. Shell thickness was measured using a digital micrometer with 0.1-mm precision (Manufacturer: Mitutoyo, Model: Type A (422), São Paulo, Brazil) on the midline of the egg; this analysis was performed after drying the shells in a 55°C forced-air oven (Manufacturer: Solab, Model: SL-102/1248-120°C, Piracicaba, Brazil). To evaluate specific gravity, eggs were immersed in different saline solutions adjusted to a volume of 25 liters of water with densities ranging from 1.060 to 1.100, at 0.0025 g/cm³ intervals.

At the end of the experiment, eight birds per treatment were randomly selected and euthanized using an electronic method. The liver, spleen, heart, and abdominal fat were then collected and weighed using an analytical balance. The results were expressed as values relative to the birds’ body weight.

### Histological and morphometric studies

#### Histological processing

The histological processing was carried out at the Histology Laboratory of the Graduate Program in Animal Science at UFPB/CCA. Biological samples of the intestine (duodenum and jejunum), liver, uterus, and kidney from 8 randomly selected animals per treatment were collected and fixed in buffered formalin for 24 h and embedded in paraffin. Tissue sections were cut at a thickness of 5 µm. Hematoxylin–eosin and Periodic Acid–Schiff (PAS) staining were used depending on the analysis, and the acquisition of digitized images was performed using an Olympus BX-60 microscope (Manufacturer: Olympus, Model: BX-60, Tokyo, Japan) and a Zeiss AxioCam camera (Manufacturer: Zeiss, Model: Axiocam 305 color, Oberkochen, Germany) coupled with the Motic Image Plus 2.0 digital image capture software.

Duodenum samples were collected 4 cm after the ventriculus; jejunum samples were collected from the middle region of this segment. Both were embedded transversely to allow visualization of intestinal villi as well as the lumen of the organ. Kidney and liver samples were collected to never exceed 0.5 cm³ in order to ensure proper tissue fixation. Uterus samples were collected from the mid-lateral region with a size of 1 cm².

#### Intestinal histomorphometry

For the histomorphometric analyses of the intestinal mucosa, 8 animals from each treatment were used, following the histological processing described above. For each animal, three photomicrographs were captured using a 5 × objective lens. In each photomicrograph, two measurements of villus height, its respective crypt, and villus width were performed, resulting in a sample size of 48 measurements per treatment for each variable, according to a modified methodology described by Moreira Filho et al. (2015).

Villus height was measured from its base to its apex; villus width was measured at the mid-portion of each villus; and crypt depth was measured from the base of the corresponding villus. The villus:crypt ratio was determined by dividing villus height by its respective crypt depth, and villus area was calculated by multiplying villus height by width, according to a modified methodology described by Moreira Filho et al. (2015).

### Hepatic glycogen score

For the analysis of hepatic glycogen storage, the Periodic Acid-Schiff (PAS) staining was used, which stains glycoproteins, including hepatic glycogen. A total of six photomicrographs per animal, resulting in a sample size of 48 per treatment (6 photomicrographs × 8 animals), were analyzed under light microscopy by the same histologist, who was blinded to the treatment groups.

The samples were classified according to the degree of glycogen deposition based on PAS staining intensity: Grade +, low hepatic glycogen deposition; Grade ++, moderate hepatic glycogen deposition; and Grade +++, high hepatic glycogen deposition. For statistical analysis of the hepatic glycogen deposition index, the grading crosses were converted into corresponding numerical values (+ = 1, ++ = 2, +++ = 3), according to the modified semi-quantitative scoring system of Ishak et al. (1995).

### Statistical analysis

The data were subjected to ANOVA using R statistical software, version 4.2.0 (R Core Team, 2022), to determine the effects of different phytase concentrations and temperature ranges on the measured variables. For variables showing significant differences (*P* < 0.05), Tukey’s test was performed to compare means across temperature levels. Additionally, regression analysis was applied to identify the optimal phytase concentration.

Y_ijkl_=μ+α_i_+β_j_+(αβ)_ij_+ϵ_ijkl_

Where:

Y_ijkl_ = response variable, μ = overall mean, α_i_ = effect of the *i* th phytase level, β_j_ = effect of the *j*-th temperature range, (αβ)_ij_ = interaction effect between the *i* th phytase level and the *j*-th temperature range; ϵ_ijkl_= random error term associated with each observation, assumed to be normally distributed with zero mean and constant variance.

When a significant effect of phytase supplementation was detected, the following model was applied:

Y_i_=β_0_+β_1_X_i_+ϵ_i_

Where:

Y_i_ = response variable for the *i* th observation, β_0_ = intercept, representing the expected value of Y when phytase level is zero (baseline), X_i_= phytase level for the *i* th observation, β_1_ = slope, indicating the effect of a one-unit increase in phytase level on the response variable, ϵ_i_ = random error term for the *i* th observation, assumed to be normally distributed with zero mean and constant variance.

## Results

### Performance

Analyzing the performance variables presented in (Table 2) feed intake, egg production, egg weight, egg mass, feed conversion ratio per egg mass, and feed conversion ratio per dozen eggs — no significant interaction was observed between temperature and phytase levels. When evaluating the isolated effect of phytase, no significant differences were detected for any of the analyzed variables. However, a significant isolated effect of temperature was observed on feed intake (*P* < 0.001). Birds maintained at 24°C and 30°C showed similar feed intake, while those exposed to 36°C had reduced feed consumption.

**Table 2:** Performance of Japanese quails fed diets containing different phytase levels and subjected to different.

Parameters	Temperature	Phytase (FTU/kg)					Mean	CV, %	*P-value*			
		0	500	1000	1500	3000			Temperature	Phytase	Temperature x Phytase	Regression
FI, g/day	24	18.931	19.701	19.596	19.484	19.849	19.512a	7.737	<0.001	0.349	0.961	NS
	30	19.705	19.978	20.309	19.997	19.407	19.879a					
	36	15.024	16.179	16.484	16.118	15.471	15.855b					
	Mean	17.887	18.619	18.796	18.533	18.242						
EP, %	24	66.013	66.749	68.071	70.074	65.365	67.254b	11.670	<0.001	0.761	0.927	NS
	30	80.549	76.297	82.280	80.740	77.122	79.398a					
	36	66.379	64.943	64.313	61.510	62.570	63.943b					
	Mean	70.980	69.330	71.555	70.775	68.352						
EW, g	24	10.560	11.109	11.058	10.875	11.103	10.941	17.034	0.300	0.390	0.317	NS
	30	10.818	10.967	10.553	10.963	10.999	10.860					
	36	9.267	9.666	12.624	9.957	9.823	10.268					
	Mean	10.215	10.581	11.412	10.599	10.642						
EM, g/day	24	6.955	7.413	7.519	7.619	7.241	7.349b	21.494	<0.001	0.447	0.721	NS
	30	8.711	8.369	8.680	8.851	8.470	8.616a					
	36	6.209	6.288	8.297	6.129	6.170	6.619b					
	Mean	7.292	7.357	8.165	7.533	7.294						
FCR-EM, Kg/kg	24	2.841	2.694	2.690	2.686	2.823	2.747a	15.070	0.003	0.961	0.909	NS
	30	2.323	2.445	2.373	2.320	2.347	2.362b					
	36	2.515	2.822	2.743	2.838	2.745	2.733a					
	Mean	2.560	2.654	2.602	2.615	2.639						
FCR-DZ, Kg/dozen	24	0.362	0.360	0.357	0.351	0.376	0.361a	13.815	<0.001	0.713	0.786	NS
	30	0.303	0.323	0.301	0.306	0.312	0.309b					
	36	0.284	0.319	0.330	0.337	0.320	0.318b					
	Mean	0.316	0.334	0.329	0.332	0.336						

(FI) Feed intake, (EP) egg production, (EW) egg weight, (EM) egg mass, (FCR-EM) feed conversion ratio per egg mass, (FCR-DZ) feed conversion ratio per dozen eggs, CV% = coefficient of variation, NS = not significant. Means followed by different letters in the columns compare temperatures within phytase levels according to Tukey’s test (*p* < 0.05).

Regarding total egg production and egg mass, temperature exerted a significant effect. Birds kept at 30°C showed higher egg production and egg mass compared to those maintained at 24°C and 36°C (*P* < 0.001). Egg weight was not significantly affected by temperature.

Feed conversion ratio per egg mass was influenced by temperature, with quails maintained at 30°C exhibiting the most efficient feed conversion compared with those at 24°C and 36°C (*P* = 0.003). For feed conversion ratio per dozen eggs, a significant effect of temperature was also detected (*P* < 0.001). Birds exposed to 30°C and 36°C had better conversion compared with those kept at 24°C.

### Egg quality

When evaluating the egg quality data (Table 3), a significant interaction between temperature and phytase levels was observed for eggshell thickness (*P* < 0.001). In the absence of phytase supplementation (0 FTU/kg), the highest eggshell thickness was recorded in birds kept at 24°C, whereas at 1500 FTU/kg, the best results were found in quails subjected to 36°C.

**Table 3:** Egg quality of Japanese quails fed diets containing different phytase levels and subjected to different temperatures.

Parameters	Temperature	Phytase (FTU/kg)					Mean	CV, %	*P-value*			
		0	500	1000	1500	3000			Temperature	Phytase	Temperature x Phytase	Regression
YC	24 30 36	5.519 5.373 5.461	5.337 5.356 5.220	5.519 5.276 5.566	5.403 5.482 5.681	5.463 5.487 5.444	5.448 5.395 5.474	4.474	0.441	0.107	0.305	NS
	Mean	5.451	5.304	5.454	5.522	5.464						
STmm	24 30 36	0.427a 0.402b 0.387b	0.406a 0.395a 0.398a	0.410a 0.411a 0.394a	0.404b 0.399b 0.443a	0.406a 0.407a 0.412a	0.411 0.403 0.407	3.602	0.118	0.033	<0.001	NS
	Mean	0.405	0.399	0.405	0.415	0.408						
SG mg/cm3	24 30 36	1.084 1.079 1.066	1.079 1.079 1.078	1.068 1.080 1.078	1.079 1.079 1.078	1.079 1.079 1.078	1.078 1.079 1.076	1.017	0.436	0.755	0.218	NS
	Mean	1.076	1.079	1.075	1.079	1.079						
HU	24 30 36	86.446 88.171 83.347	87.366 86.289 88.133	86.734 87.829 88.394	87.473 87.277 88.098	87.011 86.916 88.231	87.006 87.297 87.240	2.806	0.888	0.237	0.053	NS
	Mean	85.988	87.263	87.652	87.616	87.386						
YP%	24 30 36	32.590 30.529 32.763	30.953 31.209 31.391	31.620 32.016 32.514	32.569 31.296 31.284	31.814 31.099 32.618	31.909 31.230 32.114	5.008	0.085	0.513	0.376	NS
	Mean	31.961	31.185	32.050	31.716	31.844						
AP%	24 30 36	61.925 62.310 62.766	58.526 61.379 61.182	60.961 60.265 59.374	59.381 61.111 59.518	57.507 62.436 62.159	59.660b 61.500a 61.000ab	3.934	0.011	0.033	0.065	Q*0.013
	Mean	62.334	60.362	60.200	60.003	60.700						
SP%	24 30 36	8.076 7.896 8.126	7.908 7.596 7.595	8.011 8.012 7.816	7.907 7.753 7.633	8.053 7.877 7.704	7.991a 7.827ab 7.775b	3.902	0.021	0.012	0.666	NS
	Mean	8.033	7.700	7.946	7.764	7.878						

(YC) yolk color, (ST) shell thickness, (SG) specific gravity, (HU) Haugh unit, (YP%) yolk percentage, (AP%) albumen percentage, (SP%) shell percentage, CV% = coefficient of variation, NS = not significant; Q* = quadratic effect. Means followed by different letters in the columns compare temperatures within phytase concentrations by Tukey’s test (*p* < 0.05). *Y* = 7E-07x² − 0.0026x + 62.042; R² = 0.8701; Minimum point = 1857.143 FTU/kg.

Considering the main effects, both temperature and phytase supplementation significantly affected albumen percentage and eggshell percentage. Albumen percentage showed a quadratic response (*P* = 0.013) (Table 8), with a minimum point estimated at 1857FTU/kg of phytase; the highest values occurred in birds maintained at 30°C. Regarding eggshell percentage, quails exposed to 24°C showed higher eggshell percentages compared with those kept at 36°C (*P* = 0.021). No significant effects of treatments were observed for the remaining egg quality variables.

### Organ weights

When evaluating the organ weight variables (Table 4), a significant interaction between temperature and phytase supplementation was observed for liver weight (*P* = 0.010). At 30°C, the highest liver weights were recorded in quails fed 0, 500, and 3000 FTU/kg of phytase, while no significant differences were detected among the other phytase levels (1000 and 1500 FTU/kg) or at 24 and 36°C. For heart weight, only temperature exerted a significant effect. Birds maintained at 24°C showed a higher heart weight compared to those kept at 30 and 36°C (*P* = 0.005). No significant effects of phytase levels or temperature were observed for spleen weight and abdominal fat weight.

**Table 4:** Relative organ weights (% body weight) of Japanese quails fed diets containing different phytase levels and reared under different temperatures.

Parameters	Temperature	Phytase (FTU/kg)					Mean	CV, %	*P-Value*			
									Temperature	Phytase	Temperature x Phytase	
		0	500	1000	1500	3000						Regression
Spleen weight. %	24	0.069	0.063	0.066	0.054	0.071	0.065	2.200	0.095	0.617	0.535	NS
	30	0.078	0.074	0.072	0.063	0.075	0.073					
	36	0.049	0.045	0.042	0.077	0.077	0.058					
	Mean	0.066	0.061	0.060	0.064	0.075						
Liver weight. %	24	2.767ab	2.008b	2.411a	2.429a	2.653ab	2.454	7.500	<0.001	0.338	0.010	NS
	30	3.013a	2.814a	2.377a	2.327a	3.017a	2.710					
	36	2.149b	2.431ab	2.192a	2.166a	2.112b	2.210					
	Mean	2.643	2.418	2.327	2.308	2.594						
Heart weight. %	24	0.916	0.936	0.891	0.924	0.968	0.927a	17.328	0.005	0.873	0.747	NS
	30	0.870	0.907	0.906	0.874	0.869	0.885ab					
	36	1.010	0.884	0.820	0.814	0.843	0.874b					
	Mean	0.932	0.909	0.872	0.871	0.893						
Abdominal fat weight. %	24	0.119	0.168	0.175	0.116	0.211	0.158	9.400	0.352	0.305	0.055	NS
	30	0.135	0.050	0.029	0.226	0.250	0.138					
	36	0.135	0.229	0.027	0.107	0.066	0.113					
	Mean	0.130	0.149	0.077	0.150	0.176						

CV% = Coefficient of variation; NS = not significant. Means followed by different letters within columns compare temperatures within each phytase concentration according to Tukey’s test (*p* < 0.05).

### Duodenal intestinal morphometry

Based on the histomorphometric analysis of the duodenum (Table 5), it was observed that villus width was influenced by phytase supplementation at 30°C in a quadratic manner (*P* < 0.014), and at 36°C in a linear manner (*P* < 0.009) (Table 8). A significant temperature × phytase interaction was also detected (*P* = 0.021). When comparing phytase levels within each temperature, differences were observed only at 0 FTU/kg under 24°C, where birds showed reduced villus width compared with the other conditions.

**Table 5:** Duodenal intestinal morphometry of japanese quails fed diets containing different phytase levels and exposed to different thermal environments.

Parameters	Temperature	Phytase (FTU/Kg)					Mean	CV, %	*P-Value*			
		0	500	1000	1500	3000			Temperature	Phytase	Temperature x Phytase	Regression
Villus width (µm)	24	116.48b	115.25a	121.04a	126.23a	121.27a	120.06					NS
	30	135.33a	117.67a	118.18a	123.78a	118.52a	122.70	6.54	0.320	0.002	0.021	Q*0.014
	36	129.38a	118.53a	123.39a	121.88a	116.15a	121.86					L*0.009
	Mean	127.06	117.15	120.87	123.96	118.65						
Villus height (µm)	24	778.57b	821.35a	859.68a	789.96a	821.92a	816.09					NS
	30	820.93ab	831.20a	874.12a	781.94a	838.98a	829.43	6.73	0.480	0.009	0.002	NS
	36	892.12a	806.60a	806.12a	785.08a	793.97a	816.78					Q*0.005
	Mean	830.54	919.72	846.64	788.66	818.29						
Crypt depth (µm)	24	71.28b	75.19a	71.22a	70.63a	59.85a	69.63					Q*0.014
	30	68.56b	69.24b	71.77a	67.03ab	62.94a	67.91	7.58	0.196	<0.001	<0.001	L*0.007
	36	79.11a	65.02b	64.85a	64.29b	65.32a	67.72					Q*<0.001
	Mean	72.99	69.82	69.28	67.32	62.70						
Villus/Crypt (µm)	24	11.21	12.72	13.68	12.26	13.85	12.74					L*<0.001
	30	12.40	12.78	13.44	11.97	13.62	12.84	7.32	0.852	<0.001	0.194	L*0.039
	36	11.66	13.09	13.83	12.07	13.03	12.73					NS
	Mean	11.76	12.86	13.65	12.10	13.50						
Absorptive area (µm^2^)	24	84142b	91843a	103295a	97382a	90947b	93522					Q*<0.001
	30	112396a	98414a	103811a	91583a	102000a	1016641	8.22	<0.001	<0.001	<0.001	Q*0.001
	36	115964a	92088a	96599a	95119a	93248ab	98603					Q*0.002
	Mean	104167	94115	101235	94695	95397						

CV% = Coefficient of variation; NS = not significant. Means followed by different letters within columns compare temperatures within each phytase concentration according to Tukey’s test (*p* < 0.05).

Villus height exhibited a significant quadratic effect (*P* < 0.005) at 36°C (Table 8), along with a significant temperature × phytase interaction (*P* = 0.002). Differences among temperatures within phytase levels were detected only at 0 FTU/kg, where birds exposed to heat stress (36°C) presented higher villus height compared with those kept at 24°C and 30°C.

Crypt depth showed significant effects of phytase in all temperature conditions: quadratic at 24°C (*P* < 0.014) and 36°C (*P* < 0.001), and linear at 30°C (*P* < 0.007) (Table 8). A significant temperature × phytase interaction was also observed (*P* < 0.001). Evaluating temperature effects within each phytase level, at 0 FTU/kg the 36°C environment resulted in greater crypt depth; at 500 and 1500 FTU/kg, birds maintained at 24°C had higher crypt depth values.

A linear increasing effect of phytase on the villus height-to-crypt depth ratio (VH:CD) was observed at 24°C (*P* < 0.001) and 30°C (*P* = 0.039), with VH:CD values rising progressively as the phytase levels increased in quails maintained at these temperatures.

The absorptive area was affected by phytase supplementation at all temperatures (24, 30, and 36°C) in a quadratic manner (*P* < 0.001; *P* < 0.001; *P* < 0.002) (Table 8), with additional effects of temperature and the interaction temperature × phytase (*P* < 0.001). Within each phytase level, at 0 FTU/kg the 30°C and 36°C environments promoted greater absorptive area than 24°C. At 3000 FTU/kg, the 30°C environment yielded the highest absorptive area, while no significant differences among temperatures were observed for the remaining treatments.

### Jejunal intestinal morphometry

Based on the histomorphometric analyses of the jejunum (Table 6), villus width was influenced by both temperature and phytase supplementation. Quadratic effects were observed at 24°C (*P* < 0.0207), 30°C (*P* < 0.0377), and 36°C (*P* < 0.0001), with estimated minimum and maximum values of 650 FTU (minimum) at 24°C, 1850 FTU (maximum) at 30°C, and 1500 FTU (minimum) at 36°C, respectively (Table 8). A significant temperature × phytase interaction was also observed (*P* = 0.001). At 0 FTU of phytase, quails maintained at 24°C showed greater villus width compared with those kept at 30°C. However, at phytase levels of 1000 and 1500 FTU, quails reared at 30°C already exhibited villus width values similar to those of birds maintained at 24°C, considered a thermoneutral temperature. In contrast, at the same phytase levels (1000 and 1500 FTU), quails exposed to severe heat stress (36°C) showed lower villus width values compared with birds at 24 and 30°C.

**Table 6:** Jejunal intestinal morphometry of japanese quails fed diets with different phytase levels and exposed to different thermal environments.

Parameters	Temperature	Phytase (FTU/Kg)					Mean	CV, %	*P-Value*			
									Temperature	Phytase	Temperature x Phytase	
		0	500	1000	1500	3000						Regression
Villus width (µm)	24	87.35a	83.25a	85.00a	88.33a	91.47a	87.08	6.88	<0.001	0.002	0.001	Q*0.021
	30	80.76b	82.92a	90.14a	84.80a	85.26a	84.77					Q*0.038
	36	84.18ab	76.67a	77.49b	74.72b	86.30a	79.87					Q*<0.001
	Mean	84.10	80.95	84.21	82.62	87.67						
Villus height (µm)	24	572.18a	522.79a	504.57a	623.96a	518.53a	548.41	8.82	<0.001	0.001	<0.001	NS
	30	426.90b	462.90b	496.89a	399.65b	411.35b	439.39					L*0.025
	36	422.41b	319.40c	400.21b	406.21b	407.34b	391.12					NS
	Mean	473.83	434.79	467.22	476.61	445.74						
Crypt depth (µm)	24	55.02a	52.43a	52.73ab	61.86a	56.49a	55.71	8.93	<0.001	0.003	<0.001	NS
	30	47.80b	50.91a	59.89a	49.79b	49.99b	51.67					Q*0.002
	36	47.64b	43.84b	48.48b	47.36b	49.49b	47.36					NS
	Mean	50.16	49.06	53.70	53.01	51.99						
Villus/Crypt (µm)	24	11.43	9.99	9.98	10.67	9.68	10.35a	7.79	<0.001	<0.001	0.170	L*0.001
	30	9.98	9.39	9.57	9.17	8.99	9.42b					L*0.014
	36	10.52	8.64	8.89	9.01	8.80	9.17b					Q*0.002
	Mean	10.64	9.34	9.48	9.62	9.16						
Absorptive área (µm^2^)	24	52205a	40500a	43449a	51154a	47041a	46870	11.64	<0.001	<0.001	<0.001	NS
	30	33936b	35815a	44059a	33536b	37880b	37045					NS
	36	34912b	25628b	30240b	29573b	35813b	31233					Q*0.001
	Mean	40351	33981	39249	38088	40244						

CV% = Coefficient of variation; NS = not significant. Means followed by different letters within columns compare temperatures within each phytase concentration according to Tukey’s test (*p* < 0.05).

For villus height, both phytase and temperature effects were detected, with a linear response at 30°C (*P* < 0.025) (Table 8). There was also a significant temperature × phytase interaction (*P* < 0.001). Across all phytase levels, the highest villus height was observed in quails maintained at 24°C. Crypt depth was influenced by both phytase and temperature. A quadratic effect of phytase was observed at 30°C (*P* < 0.002), with the maximum estimated value occurring at 1925 FTU (Table 8). A significant interaction between temperature and phytase was also detected (*P* < 0.001). When treatments were compared within each phytase level, quails maintained at 24°C showed higher crypt depth values at phytase levels of 0, 500, 1500, and 3000 FTU/kg compared with those reared at 36°C. However, at the phytase level of 1000 FTU/kg, quails maintained at 30°C exhibited greater crypt depth than those exposed to 36°C.

For the villus:crypt ratio, no significant interaction between temperature and phytase supplementation was detected; however, independent effects of both factors were observed. Phytase supplementation promoted a linear decrease in the villus:crypt ratio in quails maintained at 24°C (*P* = 0.001) and 30°C (*P* = 0.014), whereas a quadratic response was observed in birds exposed to 36°C (*P* = 0.002), with the minimum estimated villus:crypt ratio occurring at 2000 FTU (Table 8). Regarding temperature, quails maintained at 24°C exhibited a higher villus:crypt ratio (*P* < 0.001) compared with those reared at 30 and 36°C.

Regarding the absorptive area, a significant temperature × phytase interaction was observed (*P* < 0.001). At phytase supplementation levels of 500 and 1000 FTU, quails maintained at 30°C exhibited a greater absorptive area compared with those exposed to 36°C, showing values similar to those observed in birds reared at 24°C at the same phytase levels. In contrast, at phytase levels of 0, 1500, and 3000 FTU, quails maintained at 24°C presented a greater absorptive area than those reared at 30 and 36°C. Additionally, regression analysis revealed a quadratic effect of phytase in quails exposed to 36°C (*P* = 0.001), with the minimum estimated absorptive area occurring at 1308 FTU (Table 8).

### Hepatic glycogen storage score

Evaluating the hepatic glycogen storage scores and the frequency distribution of scores in Japanese quail livers (Table 7), it was observed that the 24°C environment cannot be considered the most suitable thermal comfort range for these birds, as it resulted in the lowest hepatic glycogen index. In contrast, birds maintained at 30 and 36°C showed higher levels of hepatic glycogen deposition.

**Table 7:** Hepatic glycogen storage score and score frequency by treatment in japanese quail during the second laying cycle, fed diets containing phytase superdosing and exposed to different thermal environments.

Temperature	Phytase level (FTU/Kg)	1*	2*	3*	Mean/SD
24°C	0	46	2	0	1.042±0.202C**
	500	22	26	0	1.542±0.504B**
	1000	39	09	0	1.188±0.394C**
	1500	23	25	0	1.521±0.505B**
	3000	22	26	0	1.542±0.504B**
30°C	0	6	30	12	2.146±0.618A**
	500	24	24	0	1.625±0.489B**
	1000	28	20	0	1.417±0.498BC**
	1500	7	40	1	1.583±0.539B**
	3000	5	43	0	1.896±0.309AB**
36°C	0	10	28	10	2.000±0.652A**
	500	13	35	0	1.750±0.438AB**
	1000	3	45	0	1.938±0.245AB**
	1500	16	32	0	1.667±0.476B**
	3000	6	40	2	1.917±0.404AB**

SD (Standard deviation), 1 (low positivity), 2 (moderate positivity), and 3* (high/intense positivity), adapted from Ishak et al. (1995).** Frequency of the score of each photomicrograph analyzed per treatment. *P* < 0.005, ANOVA, Tukey’s post hoc test.

**Table 8:** Regression equations for egg quality parameters and duodenal and jejunal intestinal morphometry of Japanese quail fed diets containing different phytase levels and exposed to different thermal environments.

**Variables**	**Cycle**	**Effect**	**Equation**	**R^2^**	**level**
albumen percentage %	-	Quadratic	*Y* = 7E-07×^2^ - 0.0026x + 62.042	R² = 0.8701	1857 FTU ^Min^
Duodenal villus width	2	Quadratic	*Y*= −0,000003×^2^-0.0137+130.84	R^2^= 0.4911	2283 FTU ^Min^
		Linear	*Y*=−0.0034x+12.76	R^2^= 0.271	-
Duodenal villus height	2	Quadratic	*Y* = 3E-0.5×^2^-0.1079x+879.58	R^2^= 0.8739	1798 FTU ^Min^
Duodenal crypt depth	2	Quadratic	*Y*=−2E-06×^2^+0.0023x+72.294	R2= 0.9413	575 FTU ^Máx^
		Linear	*Y*=−0.0022x+70.555	R2= 0.6061	-
		Quadratic	*y* = 4E-06×^2^ - 0.016x + 76.592	R² = 0.794	2000 FTU ^Min^
Duodenal villus/Crypt	2	Linear	*Y* = 0.0004x+12.302	R^2^= 0.2949	-
Duodenal absorptive area	2	Quadratic	*Y*=−0.006×^2^+19.918+84555	R^2^= 0.8184	1660 FTU ^Máx^
		Quadratic	*Y* = 0.0053×^2^-19.009+111225	R^2^= 0.6907	1793 FTU ^Min^
		Quadratic	*y* = 0.0051×^2^ - 20.899x + 110923	R² = 0.6443	2049 FTU ^Min^
Jejunal villus width	2	Quadratic	*Y* = 1E-0.6×^2^-0.0013x+85.917	R^2^= 0.71	650 FTU ^Min^
		Quadratic	*Y*=−2E-06×^2^+0.0074x+80.931	R^2^= 0.5172	1850 FTU ^Max^
		Quadratic	*Y* = 4E-06×^2-^0.012 × 83.505	R^2^= 0.9301	1500 FTU ^Min^
Jejunal villus height	2	Linear	*Y*=−1E-05×^2^+444.09	R^2^= 0.2403	-
Jejunal crypt depth	2	Quadratic	*Y*=−2E-06×^2^+0.0077+48.612	R^2^= 0.3122	1925 FTU ^Máx^
Jejunal villus/Crypt	2	Linear	*Y* = 0.0633x+46794	R^2^= 0.0002	-
		Linear	*Y* = 0.6868x+36221	R^2^= 0.0341	-
		Quadratic	*Y* = 4E-07×^2^ - 0,0016x + 10,1	R^2^= 0,6026	2000 FTU ^Min^
Jejunal absorptive area	2	Quadratic	*Y* = 0.0026×^2^-6.803x+32804	R^2^= 0.6002	1308 FTU ^Min^

## Discussion

### Performance parameters

Although the literature reports numerous studies on the detrimental effects of heat stress on the productivity of Japanese quail (*Coturnix japonica*), this condition remains a major challenge in the poultry industry (Wasti et al., 2020). Heat stress disrupts physiological homeostasis, triggering oxidative stress, acid–base imbalance, and immunosuppression. These disturbances lead to increased mortality, reduced feed efficiency, and significant impairments in body weight, feed intake, and egg production (Ribeiro et al., 2024a).

**Fig. 2:**
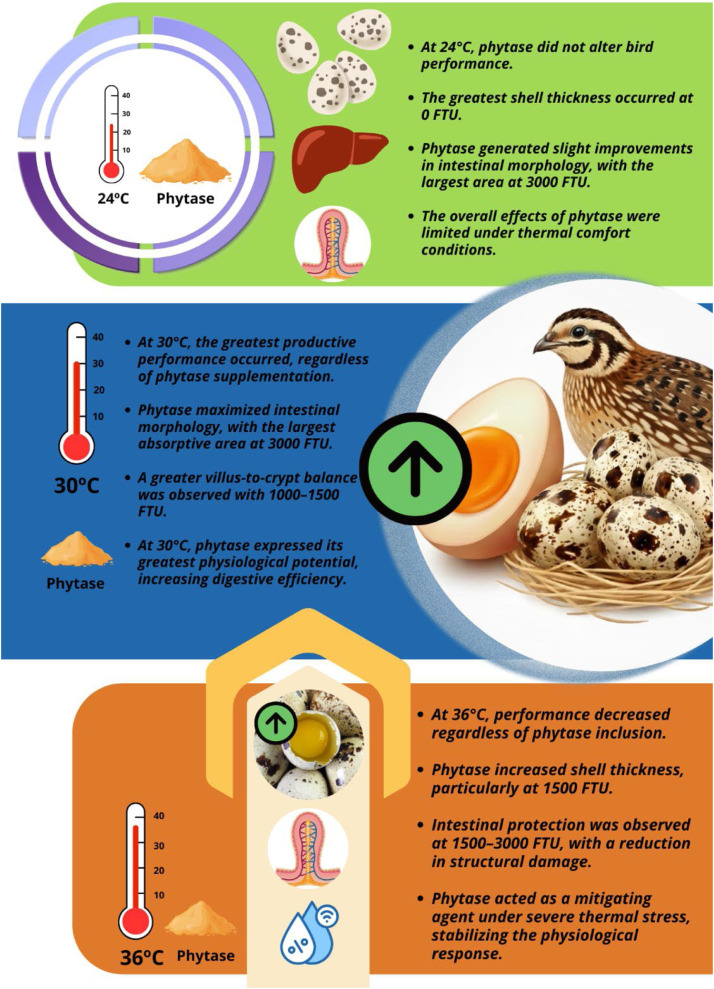
Schematic representation of the effects of phytase supplementation in Japanese quails exposed to different thermal conditions (24, 30, and 36°C), illustrating changes in productive performance, intestinal morphology, and eggshell quality across temperatures and phytase inclusion levels.

In the present study, high ambient temperatures negatively affected egg production. Exposure to severe heat stress (36°C) markedly reduced feed intake, resulting in insufficient ingestion of essential nutrients and vitamins (Fig. 2). Corroborating these findings, Ribeiro et al. (2025) reported that exposure of quails to high ambient temperatures is highly detrimental to productive performance, as birds subjected to severe heat stress (36°C) exhibited a significant reduction in feed intake, which may compromise the nutritional supply required for fundamental physiological processes, including the development and maintenance of bone tissue. This reduction in feed intake represents a common adaptive response of birds under heat stress, aiming to minimize endogenous heat production associated with digestion and nutrient metabolism (Freitas et al., 2017; Ribeiro et al., 2024a).

The effect of temperature on feed intake observed in our study is consistent with Rodrigues et al. (2022), who reported reduced feed consumption in quails exposed to 34°C. Similarly, Akdemir et al. (2019) demonstrated that Japanese quail subjected to temperatures between 29 and 34°C exhibited lower feed intake, reduced egg production and egg weight, as well as higher feed conversion ratios.

For Japanese quail, the thermoneutral zone ranges from 21°C to 27°C (Alagawany et al., 2017; Ribeiro et al., 2024a). However, under the experimental conditions of the present study, birds exposed to 30°C exhibited higher total egg production, greater egg mass, and a lower feed conversion per egg mass compared with quails maintained at 24°C and 36°C. Although 24°C falls within the thermoneutral range and does not impose thermal stress, the superior productive response observed at 30°C suggests that this temperature may create conditions that favor productive efficiency without disrupting physiological homeostasis. In this context, exposure to 30°C appears to represent a mild and well-tolerated thermal challenge, under which quail are able to maintain metabolic stability while promoting adaptive adjustments in energy partitioning toward egg production rather than solely toward body heat conservation (Lara and Rostagno, 2013).

This response may be partly explained by species-specific characteristics of laying quail, which present a more favorable surface area-to-body volume ratio compared with laying hens and broilers, enhancing their capacity to dissipate endogenous heat (Porto et al., 2021). Consequently, moderate heat exposure may be better tolerated by quail, particularly when compared with more severe heat stress conditions, such as 36°C, which are consistently associated with impaired performance. Although temperatures around 30°C exceed the ideal thermal range, quail appear capable of sustaining productive performance under these conditions, especially when nutritional strategies support the increased metabolic demands associated with thermal adaptation, including adequate vitamin and mineral supply (Truong et al., 2023).

The improved feed conversion observed in birds maintained at 30°C indicates that they utilized nutrients more efficiently for production. Under elevated temperatures, birds allocate a significant portion of their energy to heat dissipation mechanisms, which reduces the efficiency of productive metabolism. This energetic redistribution compromises nutrient utilization and worsens feed conversion. These results differ from those reported by Barros et al. (2024), who observed better feed conversion ratios in Japanese quails exposed to temperatures of 32.1°C.

On the other hand, phytase supplementation did not promote any significant effect on the evaluated performance parameters, regardless of temperature. This finding suggests that, under the experimental conditions of the present study, phosphorus availability was not the limiting factor for performance, but rather the physiological stress caused by heat. Phytase enhances the release of Ca and P bound to the phytate molecule, helping to meet the mineral requirements of birds. However, when the diet already provides sufficient calcium and phosphorus, enzymatic supplementation tends to elicit no additional response, as the released minerals exceed the physiological needs (Pongmanee et al., 2020).

### Egg quality parameters

When birds are exposed to heat stress, significant physiological changes occur that may compromise egg quality. Under such conditions, a reduction in feed intake and an increase in energy loss associated with thermoregulation are observed, resulting in lower availability of energy and nutrients for productive processes such as the formation and deposition of egg components (Castro et al., 2019).

In the present study, the egg quality indicators showed, overall, significant responses restricted to specific parameters. Shell thickness demonstrated a significant interaction between temperature and phytase supplementation. In birds that did not receive phytase, the greatest shell thickness was observed at 24°C. However, in those supplemented with 1500 FTU/kg of phytase, an increase in shell thickness was observed at 36°C, demonstrating a compensatory effect of the enzyme under heat stress conditions. Increasing dietary phytase levels exerted a positive effect on shell thickness, as a result of enhanced availability and retention of essential minerals such as calcium and phosphorus due to the hydrolysis of phytate.

Dietary supplementation with 1500 FTU/kg of phytase proved particularly effective in mitigating the deleterious effects of heat stress (36°C), improving egg quality by increasing shell thickness and strength. Ribeiro et al. (2024a), when evaluating Japanese quails exposed to three temperature ranges (24, 30, and 36°C), verified that at 30°C the birds exhibited greater efficiency in calcium absorption and utilization compared to those maintained at 24°C, possibly due to an increased metabolic demand for minerals under elevated temperatures.

Albumen and shell percentages were individually affected by both temperature and phytase supplementation. The best shell percentage was observed at 24°C, reinforcing the positive impact of thermal comfort on mineral metabolism. Albumen percentage, in turn, was favored at 30°C, indicating that intermediate temperatures may promote a balanced distribution of egg components. Kim et al. (2024), when evaluating laying hens exposed to different temperatures (21 to 33°C), observed that heat stress negatively affected all egg quality parameters.

Our results clearly demonstrate that phytase supplementation is an effective nutritional strategy to mitigate heat stress effects and optimize health, mineral metabolism, and productivity of birds under high-temperature conditions. Under heat stress, characterized by temperatures between 30 and 33°C for quails, a reduction in the expression of the calcium transporter calbindin-D28k has been observed in the ileum, cecum, colon, and uterus (Morais et al., 2021). This alteration impairs calcium absorption and deposition mechanisms, leading to the deterioration of eggshell quality in high-temperature environments (Ebeid et al., 2012).

However, Ribeiro et al. (2024a), when analyzing bone parameters and the expression of calbindin-D28k in Japanese quails supplemented with increasing phytase levels (0, 500, 1000, 1500, and 3000 FTU/kg) and subjected to heat stress (24, 30, and 36°C), observed that phytase supplementation improved calcium absorption efficiency, reflected by increased calbindin-D28k expression in the duodenum and jejunum. This response resulted in greater calcium mobilization to bone tissue (tibia) and an increase in total egg production under heat stress conditions. Despite the environmental and nutritional alterations imposed by the treatments, internal egg quality remained unaffected, as specific gravity, yolk color, and Haugh Unit were not statistically influenced. This outcome may be explained by the fact that internal quality parameters are predominantly associated with albumen protein integrity and dietary pigment supply, rather than mineral availability. Phytase supplementation primarily enhances calcium and phosphorus utilization, exerting a direct effect on eggshell formation, but showing limited influence on albumen height or yolk pigmentation. Moreover, the duration and intensity of heat stress applied in the present study may not have been sufficient to promote protein denaturation or oxidative alterations capable of impairing these internal egg quality traits.

### Organ weight

In our study, the assessment of the relative weight of organs revealed that only the liver was sensitive to the interaction between temperature and phytase supplementation. When treatments were compared within each phytase level, birds maintained at 30°C generally exhibited higher relative liver weight than those exposed to 36°C, particularly at 0 and 3000 FTU, while birds kept at 24°C showed intermediate values. This pattern indicates that moderate thermal conditions at 30°C supported greater hepatic metabolic activity, which is consistent with the superior productive performance observed at this temperature, rather than reflecting a pathological metabolic overload. In contrast, the reduction in liver weight at 36°C suggests metabolic suppression associated with severe heat stress, likely resulting from reduced feed intake and endocrine adjustments aimed at minimizing endogenous heat production. Similar responses have been reported by Barros et al. (2024), who observed that exposure of Japanese quail to high ambient temperatures led to reductions in the relative weights of metabolically active organs, including the liver, as a consequence of decreased metabolic rate and hormonal modulation, particularly reductions in thyroid hormones (T3 and T4).

The liver plays a central role in energy, lipid, and mineral metabolism in laying birds, particularly in the synthesis of lipoproteins required for yolk formation. Therefore, increases in liver mass under productive conditions may reflect enhanced metabolic activity to support egg production. Conversely, under severe heat stress, the body prioritizes survival mechanisms, reducing the mass and activity of internal organs as an adaptive strategy to lower maintenance energy requirements, as previously described for quail and broilers exposed to elevated temperatures (Oba et al., 2012; Barros et al., 2024). In this context, liver mass can also serve as an indirect indicator of nutritional modulation, including improved mineral utilization in birds receiving phytase.

Maia et al. (2025), when evaluating Japanese quails during the second laying cycle supplemented with phytase superdoses and subjected to different temperatures (24, 30, and 36°C), reported a reduction in liver weight with increasing phytase levels up to approximately 2000 FTU, which was attributed to improved nutrient efficiency resulting from phytate hydrolysis. However, the present results indicate that the effects of phytase on liver weight are highly dependent on the thermal environment and physiological status of the birds. Notably, at phytase levels of 1000 and 1500 FTU, no differences in liver weight were observed among temperatures, suggesting that phytase supplementation may attenuate the negative effects of thermal stress on hepatic metabolism by improving nutrient utilization efficiency and reducing metabolic variability across environments.

Another important organ evaluated was the heart. In the present study, birds maintained at 24°C showed higher relative heart weight compared with those exposed to 30°C and 36°C. As ambient temperature increases, birds activate physiological mechanisms to reduce endogenous heat production, including decreased basal metabolic rate and alterations in endocrine regulation. These responses involve increased circulating corticosterone concentrations and a concomitant reduction in thyroid hormones triiodothyronine (T3) and thyroxine (T4), which are key regulators of metabolic intensity and organ growth. Although these adjustments contribute to thermal adaptation, they also result in reduced development of metabolically active organs.

Supporting this interpretation, Lima et al. (2025) observed increased corticosterone concentrations in Japanese quails subjected to severe heat stress (36°C) and fed phytase-superdosed diets. In the present study, higher circulating T4 relative to T3 during the second production cycle further indicates a downregulation of metabolic activity under high-temperature conditions. Together, these endocrine responses represent adaptive strategies to reduce endogenous heat production, which may explain the lower weights of internal organs, particularly under severe heat stress at 36°C.

### Intestinal morphometry of the duodenum and jejunum

The integrity of the intestinal barrier is essential for health, acting in selective permeability for nutrients and in restricting pathogenic entry (Greene et al., 2025). However, heat stress compromises this vital structure, as increased environmental temperature redirects visceral blood flow (a reduction of 30–50%) toward the body surface. This cooling mechanism leads to intestinal ischemia and hypoxia, weakening the barrier (Fan et al., 2025).

In the present study, duodenal intestinal morphology responded markedly to the interaction between temperature and phytase supplementation. The greatest villus height and width were observed in birds without enzymatic supplementation maintained at 30 and 36°C. At 30°C, increased villus dimensions are indicative of enhanced absorptive surface and preserved intestinal functionality, which is consistent with the superior productive performance observed under this mild and well-tolerated thermal condition. In contrast, at 36°C, although villus height and width were increased, these changes should be interpreted cautiously, as they may reflect transient epithelial remodeling or compensatory hyperplasia in response to thermal injury rather than an improvement in absorptive efficiency.

Crypt depth also showed a significant interaction, with the greatest values observed at 36°C in the absence of phytase, suggesting intensified epithelial turnover associated with heat-induced intestinal stress. At 24°C, phytase supplementation at 500 and 1500 FTU increased crypt depth, likely reflecting a stimulatory effect on epithelial cell proliferation rather than intestinal injury. When interpreted together with the villus height and villus: crypt ratio, these responses suggest a regulated process of mucosal renewal under thermoneutral conditions.

These findings indicate that phytase contributes to maintaining intestinal integrity under milder temperatures by promoting a balance between cell renewal and absorptive capacity. Additionally, phytate hydrolysis by phytase releases minerals and nutrients otherwise bound in the phytate matrix. This synergistic effect improves both bone parameters and intestinal morphology (Ptak et al., 2015; Borda-Molina et al., 2016). In the present study, the villus: crypt ratio was affected solely by phytase, indicating improved absorptive efficiency with enzyme inclusion. Total absorptive area responded to the interaction between temperature and phytase, being higher in birds kept at 30 and 36°C without supplementation, and at 30°C with 3000 FTU. Deep crypts are indicative of accelerated cell turnover in response to epithelial injury, resulting in reduced villus:crypt ratio (V:C) (Ducatelle et al., 2018). Higher phytase doses may improve this ratio and, consequently, absorptive function (Paiva et al., 2014).

In the jejunum, morphometric changes followed a pattern similar to the duodenum but demonstrated more consistent responses to the combined effects of temperature and phytase. Villus height and width were greater in birds without supplementation at 24°C, and in birds receiving 1000 and 1500 FTU at both 24 and 36°C. These results indicate that thermoneutral environments and moderate phytase supplementation favor intestinal mucosa development and integrity. Efficient nutrient absorption depends on the villi, which are densely populated with transport proteins. However, heat stress compromises villus structure, leading to reduced absorptive area and impaired nutrient uptake (Fan et al., 2025).

Crypt depth also exhibited significant interaction: the best results occurred at 24°C at the 0, 1500, and 3000 FTU levels, and at 30°C at 1000 FTU, suggesting that the balance between cell renewal and maturation depends on both temperature and enzyme level. The villus:crypt ratio was independently influenced by phytase and temperature, with superior morphologic performance at 24°C, reinforcing the importance of thermal comfort for optimal absorptive efficiency. Previous studies showed that broilers exposed to chronic heat stress for six days presented notable duodenal and jejunal mucosal damage, resulting in reduced villus height (Mazzoni et al., 2022). Birds with longer villi tend to have higher absorptive capacity due to the presence of mature enterocytes. Other studies have reported decreased villus height and increased crypt depth in response to heat stress (Deng et al., 2012; Santos et al., 2015).

Martinez-Vallespían (2025) evaluated broiler intestinal morphometry from 1 to 21 days of age and observed no significant effect on crypt depth; however, a linear effect was found for the villus:crypt ratio with increasing phytase levels. Similarly, in our study, a linear response was also detected for jejunal V:C ratio at 24 and 30°C. Total absorptive area also responded to temperature × phytase interaction, being superior at 24°C for most supplementation levels and slightly increased at 30°C with intermediate doses (500 and 1000 FTU). As the primary site of absorption in the small intestine, jejunal morphology is essential. Taller villi and a greater V:C ratio indicate enhanced absorptive ability due to increased enterocyte maturity and surface area (Xu et al., 2003; Sen et al., 2012; Nari et al., 2020). Conversely, deeper crypts are associated with accelerated turnover and epithelial injury.

Overall, phytase proved effective in maintaining digestive and absorptive capacity under favorable thermal conditions; however, extreme heat appears to override these benefits, reducing the morphofunctional response of the intestine.

### Hepatic glycogen score

In the present study, birds exposed to 30 and 36°C without phytase supplementation exhibited higher hepatic glycogen scores. This response, however, appears to be mediated by distinct physiological mechanisms depending on the thermal environment. At 30°C, a condition previously characterized as thermally tolerable and associated with optimal productive performance in Japanese quail (Maia et al., 2025; Ribeiro et al., 2025), increased hepatic glycogen likely reflects preserved metabolic functionality and adequate glucose availability to sustain the high energy demands associated with egg production. Under this mild and well-tolerated thermal challenge, hepatic glycogen storage may represent an adaptive energy reserve rather than an indicator of metabolic impairment.

In contrast, under severe heat stress at 36°C, elevated hepatic glycogen should not be interpreted as improved hepatic efficiency. Heat stress is known to disrupt hepatic metabolism, induce oxidative damage, and alter the endocrine regulation of glucose homeostasis (Lu et al., 2019; Ding et al., 2023). According to Ma et al. (2021), chronic heat exposure elevates circulating corticosterone levels, stimulating hepatic gluconeogenesis through the upregulation of key regulatory enzymes such as phosphoenolpyruvate carboxykinase (PCK), pyruvate carboxylase (PC), and fructose-1,6-bisphosphatase (FBP1), while promoting muscle protein catabolism to provide amino acid substrates. Within this metabolic context, heat stress may favor glucose production and alter energy partitioning. Therefore, the higher hepatic glycogen score observed at 36°C in the present study likely reflects altered glycogen turnover and compromised energy mobilization under severe heat stress, rather than enhanced metabolic performance.

Interestingly, Moraes et al. (2021) reported that Japanese quail exposed to different ambient temperatures did not exhibit marked depletion of hepatic glycogen, suggesting that glycogen stability or accumulation under thermal challenge may represent a compensatory metabolic response rather than a direct indicator of hepatic dysfunction. However, in the present study, the elevated hepatic glycogen score observed specifically at 36°C appears to reflect compromised hepatic energy metabolism and altered glucose handling under severe heat stress, consistent with a stress-induced disruption of energy utilization rather than an adaptive response.

## Conclusion

Phytase supplementation promoted relevant physiological benefits in Japanese quail, particularly under thermal challenge. Although productive performance was not directly affected, supplementation at 1500 FTU/kg mitigated the detrimental effects of severe heat stress (36°C) and increased eggshell thickness, thereby improving egg quality and highlighting the role of phytase in enhancing calcium and phosphorus utilization under high-temperature conditions. In addition, intermediate phytase levels (around 1000 FTU/kg) positively modulated intestinal morphometric traits under thermoneutral (24°C) and moderately elevated temperatures (30°C), indicating improved absorptive morphology and intestinal functionality. Collectively, these findings support the use of 1500 FTU/kg of phytase as a targeted nutritional strategy under severe heat stress, while lower inclusion levels may be sufficient to support intestinal responses under thermoneutral or moderately warm environments.

## Disclosures

The authors declare that they have no known competing financial or personal interest that could have impact the outcome of the study reported in this paper.

## CRediT authorship contribution statement

**Raiane dos Santos Silva:** Writing – review & editing, Writing – original draft, Supervision, Methodology, Investigation, Formal analysis, Data curation, Conceptualization. **Apolônio Gomes Ribeiro:** Writing – review & editing, Writing – original draft, Investigation, Formal analysis, Data curation, Conceptualization. **Adiel Vieira de Lima:** Data curation, Conceptualization. **Paloma Eduarda Lopes de Souza:** Data curation, Conceptualization. **Edijanio Galdino da Silva:** Formal analysis, Data curation, Conceptualization. **Isabelle Naemi Kaneko:** Formal analysis, Data curation, Conceptualization. **Cleber Franklin Santos de Oliveira:** Data curation, Conceptualization. **Carlos Henrique do Nascimento:** Data curation, Conceptualization. **Dayane Albuquerque da Silva:** Data curation, Conceptualization. **Fernando Guilherme Perazzo da Costa:** Writing – original draft, Methodology, Funding acquisition, Formal analysis, Data curation, Conceptualization. **Edilson Paes Saraiva:** Writing – review & editing, Methodology, Formal analysis, Data curation, Conceptualization. **Lucas Rannier Ribeiro Antonino Carvalho:** Writing – review & editing, Formal analysis, Data curation, Conceptualization. **Ricardo Romão Guerra:** Writing – review & editing, Writing – original draft, Visualization, Validation, Supervision, Resources, Project administration, Methodology, Investigation, Funding acquisition, Formal analysis, Data curation, Conceptualization.

## Disclosures

The authors declare that they have no other conflicts of interest.

## References

[bib0001] Alagawany M., Farag M.R., El-Hack M.E.A.B.D., Patra A. (2017). Heat stress: effects on productive and reproductive performance of quail. World. Poult. Sci. J..

[bib0002] Akdemir F., Köseman A., Şeker I. (2019). *Alchemilla vulgaris* effects on egg production and quality expressed by heat-stressed quail during the late laying period. S. Afr. J. Anim. Sci..

[bib0003] Barros H.S.S., Oliveira R.F., Minafra C.S., Gomide A.P.C., Araujo Neto F.R., Gonçalves J.C.R., Queiroz F.H.S., Nobre G.M., Vilarinho B.D.R.S., Lima M.C., Assis S.D., Santos F.R. (2024). Functional oil in the feeding of heat-stressed Japanese quail. Poult. Sci..

[bib0004] Bernardes R.D., Oliveira C.H., Calderano A.A., Ferreira R.S., Dias K.N.M., Almeida B.F., Aleixo P.E., Albino L.F.T. (2022). Effect of phytase and protease combination on performance, metabolizable energy, and amino acid digestibility of broilers fed nutrient-restricted diets. Rev. Bras. Zootec..

[bib0005] Borda-Molina D., Vital M., Sommerfeld V., Rodehutscord M., Camarinha-Silva A. (2016). Insights into the intestinal microbiota of chickens fed diets supplemented with phosphorus, calcium, and phytase. Front. Microbiol..

[bib0008] Castro F.L.S., Kim H.Y., Hong Y.G., Kim W.K. (2019). The effect of total sulfur amino acid levels on growth performance, egg quality, and bone metabolism in laying hens subjected to high environmental temperature. Poult. Sci..

[bib0009] Cowieson A.J., Acamovic T., Bedford M.R. (2006). Supplementation of corn–soy-based diets with an *Escherichia coli*-derived phytase: effects on broiler chick performance and the digestibility of amino acids and metabolizability of minerals and energy. Poult. Sci..

[bib0010] Cowieson A.J., Wilcock P., Bedford M.R. (2011). Super-dosing effects of phytase in poultry and other monogastrics. Worlds Poult. Sci. J..

[bib0011] Dallmann H.M., Avila V.S., Krabbe E.L., Surek D., Bedendo G.C., Toledo T.S., Dallmann P.R., Roll A.A.P., Roll V.F.B., Rutz F. (2023). Different phytase levels and energy densities in broiler diets on performance, nutrient digestibility, and bone integrity from 28 to 35 days of age. Arq. Bras. Med. Vet. Zootec..

[bib0012] Dersjant-Li Y., Bello A., Stormink T., Abdollahi M.R., Ravindran V., Babatunde O.O., Adeola O., Toghyani M., Liu S.Y., Selle P.H., Marchal L. (2022). Modeling improvements in ileal digestible amino acids by a novel consensus bacterial 6-phytase variant in broilers. Poult. Sci..

[bib0013] Ding K.N., Lu M.H., Guo Y.N., Liang S.S., Mou R.W., He Y.M., Tang L.P. (2023). Resveratrol alleviates hepatic oxidative damage induced by chronic heat stress in broilers by activating the Nrf2–Keap1 signaling pathway. Ecotoxicol. Environ. Saf..

[bib0014] Ducatelle R., Goossens E., Meyer F., Eeckhaut V., Antonissen G., Haesebrouck F., Immerseel F.V. (2018). Biomarkers for monitoring intestinal health in poultry: current status and future perspectives. Vet. Res..

[bib0015] Ebeid T.A., Suzuki T., Sugiyama T. (2012). High ambient temperature influences eggshell quality and calbindin D28k localization in the eggshell gland and all intestinal segments of laying hens. Poult. Sci..

[bib0017] Gonzalez-Rivas P.A., Chauhan S.S., Ha M., Fegan N., Dunshea F.R., Warner R.D. (2020). Effects of heat stress on animal physiology, metabolism, and meat quality: a review. Meat Sci.

[bib0018] Fan X., Tian X., Li M. (2025). Challenges of heat stress on intestinal health in pig husbandry. Fundam. Res..

[bib0019] Farias M.R.S., Leite S.C.B., Silva H.P., Pacheco D.B., Alves G.C., Abreu C.G., Freitas E.R. (2021). Superdosing phytases in the diets of light laying hens: productive performance and bone quality. Braz. J. Poult. Sci..

[bib0020] Freitas H.B., Nascimento K.M.R.S., Kiefer C., Oliveira M.S., Macie V.A., Silva L.A.R., Chaves N.R.B., Flores B.D.S.C. (2017).

[bib0021] Lima L.A.A., Maia M.I.L., Afo D.I., Maia A.F., Givisiez P.E., Guerra R.R., Saraiva E.P. (2025). Effects of exogenous phytase supplementation on the modulation of hormonal responses in Japanese quails (*Coturnix japonica*) at high environmental temperatures during the production phase. Poult. Sci..

[bib0022] Mazzoni M., Zampiga M., Clavenzani P., Lattanzio G., Tagliavia C., Sirri F. (2022). Effect of chronic heat stress on gastrointestinal histology and expression of feed intake-regulatory hormones in broiler chickens. Animal.

[bib0023] Greene E.S., Roach B., Cuadrado M.F., Orlowski S., Dridi S. (2025). Effect of heat stress on ileal epithelial barrier integrity in broilers divergently selected for high and low water efficiency. Front. Phys..

[bib0025] Kim H.R., Ryu C., Lee S.D., Cho J.H., Kang H. (2024). Effects of heat stress on the laying performance, egg quality, and physiological response of laying hens. Animals.

[bib0026] Lara L.J., Rostagno M.H. (2013). Impact of heat stress on poultry production. Animals.

[bib0027] Lu Z., He X., Ma B., Zhang L., Li J., Jiang Y., Gao F. (2019). Dietary taurine supplementation decreases fat synthesis by suppressing liver X receptor alpha pathway and alleviates lipid accumulation in the liver of chronically heat-stressed broilers. J. Sci. Food Agric..

[bib0028] Ma B., Zhang L., Li J., Xing T., Jiang Y., Gao F. (2021). Heat stress alters muscle protein and amino acid metabolism and accelerates liver gluconeogenesis for energy supply in broilers. Poult. Sci..

[bib0029] Maia A.F., Ribeiro A.G., Silva R.D.S., Silva E.G.D., Lima L.A.D.A., Saraiva E.P., Queiroga F.L.P.G., Ferreira A.C.S., Rousseau X., Costa F.G.P., Guerra R.R. (2025). Egg production and biochemical evaluation of laying quails fed diets containing phytase overdosage under different thermal conditions. Animals.

[bib0031] Martínez-Vallespín B., Ader P., Zentek J. (2025). Effect of increasing levels of phytase on performance, prececal nutrient digestibility, intestinal mucosa physiology and immune response in broiler chickens from 1 to 21 days of age. Poult. Sci..

[bib0033] Moraes L.R., Delicato M.E.A., Cruz A.S., Silva H.T.F.N.P., Alves C.V.B.V., Campos D.B., Saraiva E.P., Costa F.G.P., Guerra R.R. (2021). Methionine supplementing effects on intestine, liver and uterus morphology, and on positivity and expression of Calbindin-D28k and TRPV6 epithelial calcium carriers in laying quail in thermoneutral conditions and under thermal stress. Plos One.

[bib0037] Nari N., Ghasemi H.A., Hajkhodadadi I., Farahani A.K. (2020). Intestinal microbial ecology, immune response, stress indicators, and intestinal morphology of male broilers fed low-phosphorus diets supplemented with phytase, butyric acid, or *Saccharomyces boulardii*. Livest. Sci..

[bib0038] Oba A., Lopes P.C.F., Boiago M.M., Silva A.M.S., Montassier H.J., Souza P.A. (2012). Productive and immunological traits of broiler chickens fed diets supplemented with chromium, reared under different environmental conditions. R. Bras. Zootec..

[bib0039] Paiva D., Walk C., McElroy A. (2014). Dietary effects of calcium, phosphorus, and phytase on poultry performance, intestinal morphology, mineral digestibility, and bone ash during a natural necrotic enteritis episode. Poult. Sci..

[bib0040] Ptak A., Bedford M.R., Świątkiewicz S., Żyła K., Józefiak D. (2015). Phytase modulates ileal microbiota and improves growth performance of broiler chickens. Plos One.

[bib0041] Pongmanee K., Křhn I., Korver D.R. (2020). Effects of phytase supplementation on shell and bone quality and phosphorus and calcium digestibility in laying hens from 25 to 37 weeks of age. Poult. Sci..

[bib0042] Porto M.L., Teófilo T.S., Cavalcanti D.M.L.P., Freitas C.I.A., Oliveira M.F., Fontenele-Neto J.D. (2021). Incubation variables, performance, and morphometry of the duodenal mucosa of Japanese quails (Coturnix coturnix japonica) submitted to different incubation temperatures and thermally challenged after hatching. Arqu. Bras. Med. Vet. Zootec..

[bib0044] Ribeiro A.G., Silva R.S., Costa F.S., Silva E.G., Ribeiro J.E.S., Saraiva E.P., Costa F.G.P., Guerra R.R. (2024). Phytase super-dosing modulates bone parameters and the concentration of the calcium epithelial carrier calbindin-D28k in quails (*Coturnix japonica*) under thermal stress. Anim. Prod. Sci..

[bib0045] Ribeiro A.G., Silva R.S., Costa F.S., Silva E.G., Santos Ribeiro J.E., Saraiva E.P., Costa F.G.P., Guerra R.R. (2024). Heat stress in Japanese quails (Coturnix japonica): benefits of phytase supplementation. Animal.

[bib0046] Ribeiro A.G., Silva R.S., Alves C.V.B.V., Campos D.B., Silva D.A., Nascimento J.C.S., Silva E.G., Saraiva E.P., Costa F.G.P., Pereira W.E., Carvalho L.R.R.A., Guerra R.R. (2025). Gene expression of calcium transporters Calbindin-D28K and TRPV6 in Japanese quails (Coturnix japonica) subjected to phytase super-dosing and under different temperatures. Poult. Sci..

[bib0047] Rostagno M.H. (2020). Efeitos do estresse térmico na saúde intestinal das aves. Rev. Acad. Ciênc. Anim..

[bib0048] Rostagno H.S., Albino L.F.T., Hannas M.I., Donzele J.L., Sakomura N.K., Perazzo F.G., Saraiva A., Teixeira M.L., Rodrigues P.B., de Oliveira R.F., de S.L., Barreto T., Brito C.O. (2017). https://edisciplinas.usp.br/pluginfile.php/4532766/mod_resource/content/1/Rostagno%20et%20al%202017.pdf.

[bib0049] Core Team R. (2022). https://www.R-project.org/.

[bib0050] Sampath V., Gao S., Park J.H., Kim I.H. (2023). Exogenous phytase improves growth performance, nutrient retention, tibia mineralization, and breast meat quality in Ross-308 broilers. Agriculture.

[bib0052] Santos R.R., Awati A., Roubos-van den Hil P.J., Tersteeg-Zijderveld M.H., Koolmees P.A., Fink-Gremmels J. (2015). Quantitative histo-morphometric analysis of heat-stress-related damage in the small intestines of broiler chickens. Avian Pathol..

[bib0053] Sen S., Ingale S.L., Kim Y.W., Kim J.S., Kim K.H., Lohakare J.D., Kim E.K., Kim H.S., Ryu M.H., Kwon I.K., Chae B.J. (2012). Effect of *Bacillus subtilis* LS 1–2 supplementation in broiler diets on growth performance, nutrient retention, cecal microbiology, and small intestinal morphology. Res. Vet. Sci..

[bib0054] Sena T.L., Leite S.C.B., Farias M.R.S., Abreu C.G., Freitas E.R., Costa A.C. (2020). Phytase superdosing in the diet of lightweight replacement pullets: performance, organ biometry and bone characteristics. Braz. J. Poult. Sci..

[bib0056] Truong L., Miller M.R., Sainz R.D., King A.J. (2023). Changes in Japanese quail (Coturnix coturnix japonica) blood gases and electrolytes in response to multigenerational heat stress. PLoS Clim..

[bib61] Wasti S., Sah N. (2020). Impact of heat stress on poultry health and performances, and potential mitigation strategies. Animal.

[bib0058] Xu Z.R., Hu C.H., Xia M.S., Zhan X.A., Wang M.Q. (2003). Effects of dietary fructooligosaccharide on digestive enzyme activities, intestinal microflora, and morphology of male broilers. Poult. Sci..

[bib59] Moreira Filho, A.L.B., C.J.B. Oliveira, H.B. Oliveira, D.B. Campos, R.R. Guerra, F.G.P. Costa, P.E.N. Givisiez. 2015. High incubation temperature and threonine dietary level improve ileum response against Post-Hatch salmonella enteritidis inoculation in broiler chicks. PlosOne. 10, 1-13. https://doi.org/10.1371/journal.pone.0131474. 10.1371/journal.pone.0131474 PMC4488937 26131553

[bib60] Ishak, K.,A. Baptista, L. Bianchi, F. Callea, J.D. Groote, F. Gudat, H. Denkg, V. Desmeth, G. Korbi, R.N.M. MacSweenj, M.J. Phillipsk, B.G. Portmannl, H. Poulsenm, P.J. Scheuer, M. Schmidn, H, Thalero. 1995. Histological grading and staging of chronic hepatitis. J. Hepatol. 22, 696–699. Available at: https://www.journal-of-hepatology.eu/article/0168-8278(95)80226-6/abstract. 10.1016/0168-8278(95)80226-6 7560864

